# *Helicobacter pylori* infection status had no influence on upper gastrointestinal symptoms: a cross-sectional analysis of 3,005 Japanese subjects without upper gastrointestinal lesions undergoing medical health checkups

**DOI:** 10.1007/s10388-017-0573-9

**Published:** 2017-03-15

**Authors:** Tomomi Yoshioka, Eri Takeshita, Yasuhisa Sakata, Megumi Hara, Kayo Akutagawa, Natsuko Sakata, Hiroyoshi Endo, Takashi Ohyama, Keiji Matsunaga, Yuichiro Tanaka, Shinpei Shirai, Yoichiro Ito, Nanae Tsuruoka, Ryuichi Iwakiri, Motoyasu Kusano, Kazuma Fujimoto

**Affiliations:** 1 Department of Internal Medicine, Saga Medical School, 5-1-1 Nabeshima, Saga, 849-8501 Japan; 2 Department of Gastrointestinal Endoscopy, Saga Medical School, Saga, Japan; 3 Department of Preventive Medicine, Saga Medical School, Saga, Japan; 4 Yuaikai Oda Hospital, Kashima, Saga Japan; 5 Saiseikai Karatsu Hospital, Karatsu, Saga Japan; 6 Takagi Hospital, Okawa, Fukuoka Japan; 7 Department of Endoscopic Surgery, Gunma University Hospital, Maebashi, Japan

**Keywords:** Reflux esophagitis, Gastroesophageal reflux disease (GERD), Reflux symptoms, Functional dyspepsia, Frequency scale for the symptoms of GERD (FSSG)

## Abstract

**Background:**

This study aimed to evaluate the influence of *Helicobacter pylori* infection and its eradication on the upper gastrointestinal symptoms of relatively healthy Japanese subjects.

**Methods:**

A total of 3,005 subjects (male/female: 1,549/1,456) undergoing medical health checkups were enrolled in the present study, at five hospitals in Saga, Japan, from January to December 2013. They had no significant findings following upper gastrointestinal endoscopy. All subjects completed a questionnaire that addressed a frequency scale for symptoms of gastroesophageal reflux disease. The questionnaire comprised seven questions regarding reflux symptoms and seven regarding acid-related dyspepsia, which were answered with a score based on the frequency of symptoms. *Helicobacter pylori* infection was identified by a rapid urease test and/or *H. pylori* antibody titer, and an eradication history was confirmed by the subjects’ medical records.

**Results:**

*Helicobacter pylori* infection was positive in 894 subjects out of 3,005 (29.8%). Eradication of *Helicobacter pylori* was successfully achieved in 440 subjects of 458 treated. *Helicobacter pylori* infection had no influence on the acid-related dyspepsia evaluated by the questionnaire, whereas the mean reflux score was relatively high in the *Helicobacter pylori* native negative subjects compared to *Helicobacter pylori* native positive. Eradication of *Helicobacter pylori* and time span after the eradication had no effect on the upper gastrointestinal symptoms evaluated by the questionnaire.

**Conclusion:**

*Helicobacter pylori* infection and history of eradication did not affect acid-related dyspepsia symptoms in Japanese healthy subjects.

## Introduction

An annual survey on public health conducted by the Ministry of Health, Labour and Welfare of Japan in 2013 indicated that 27.68% of males and 31.24% of females had some clinical symptoms regarding the gastrointestinal tract [1]. Several studies in Western countries indicated a relationship between *Helicobacter pylori* infection and clinical symptoms of the upper gastrointestinal tract, while the beneficial effect of *H. pylori* eradication on these symptoms was complex [2–4]. Improvement of upper gastrointestinal tract symptoms after *H. pylori* eradication is still unclear in Japan [5–12], although several prospective randomized studies in Asia and South America showed the efficacy of eradication therapy [13, 14]. These reports indicate that a relationship between dyspepsia symptoms and *H. pylori* infection is still controversial.

Although previous studies indicated that reflux esophagitis was developed following eradication therapy [4, 15], recent studies in Japan have indicated that the frequency or severity of this esophagitis, evaluated by endoscopy, was not serious [16–20]. Evaluation of reflux symptoms after eradication can be difficult, and one study in Japan suggested that the reflux symptoms might be improved and/or not exacerbated after eradication [21].

The aims of the present cross-sectional study were to determine: (1) whether reflux symptoms and acid-related dyspepsia were different between *H. pylori* positive and negative subjects; and (2) whether these symptoms were influenced by eradication of *H. pylori*. Using the modified frequency scale for symptoms of gastroesophageal reflux disease (FSSG) questionnaire [22, 23], upper gastrointestinal symptoms were evaluated in relatively healthy subjects presenting for medical health checkups, with no lesion revealed by upper gastrointestinal endoscopy.

## Patients and methods

A total of 3,505 subjects (male/female: 1,922/1,583) received upper gastrointestinal endoscopy during medical health checkups at five hospitals in Saga, Japan, from January to December 2013, as described in a previous study [24]. Written informed consent was obtained from all subjects. Among the subjects, 500 were excluded from the present analysis: 395 subjects with endoscopic reflux esophagitis (graded according to the Los Angeles classification [25]; 27 with gastric cancer and/or gastric ulcers; 24 with duodenal ulcers; and 54 who were prescribed with anti-acid medicines (histamine-2 receptor antagonists or proton pump inhibitors), and/or prokinetics. Thus, 3,005 subjects were enrolled in the present analysis, all with no lesions which could lead to upper gastrointestinal symptoms. *H. pylori* infection was identified by a rapid urease test and/or *H. pylori* antibody titer, and an eradication history was confirmed by the subject’s medical record and medical history form to confirm the time span after eradication. The successfully eradicated subjects were divided into two groups: the time span was more than 3 years and less than 3 years.

All subjects completed a modified FSSG questionnaire, which is a self-administered, validated questionnaire comprising 14 questions, with seven regarding reflux symptoms and seven regarding acid-related dyspepsia [22]. Each symptom is assigned a score [never experienced = 0; occasionally (30% of the time) = 1; sometimes (50%) = 2; often (70%) = 3; and always (100%) = 4]. The seven questions about reflux symptoms are: Q1, “Do you get heartburn?”; Q2, “Do you sometimes subconsciously rub your chest with your hand?”; Q3, “Do you get heartburn after meals?”; Q4, “Do you have an unusual (e.g. burning) sensation in your throat?”; Q5, “Do some things get stuck when you swallow?”; Q6, “Do you feel a bitter liquid (acid) coming up into your throat?”; and Q7, “Do you get heartburn if you bend over?”. The seven questions about acid-related dyspepsia are: Q8, “Does your stomach get bloated?”; Q9, “Does your stomach ever feel heavy after meals?”; Q10, “Do you feel sick after meals?”; Q11, “Do you feel full while eating meals?”; Q12, “Do you burp a lot?”; Q13, “Do you feel pain in the upper abdomen after meals?”; Q14, “Do you feel pain in the upper abdomen while fasting?”.

All procedures performed in this study were approved by the Ethical Committee of Saga University Hospital (2014–09–15). Statistical evaluation was carried out using the *χ* ^2^ test and Welch’s *t* test (using SPSS software, version 22; SPSS, Tokyo, Japan), and statistical significance was established at a *p* value of <0.05.

## Results

Table 1 shows the background characteristics of all 3,005 subjects enrolled in the present study. Their average age was 54.2 years, and the numbers of male and female subjects were almost equal. The comorbidity rate of hiatus herniation was 29.6% (894/3,005). The rate of short segment Barrett’s esophagus was relatively high (23.7%), whereas the rate of long segment Barrett’s esophagus was very low (0.3%). Gastric ulcer scars and duodenal ulcer scars were detected by endoscopy only in 3.0 and 2.6% of subjects, respectively. The rate of *H. pylori* infection was 29.8% (894/3,005: native positive) at the time of the subjects’ medical health checkups. A total of 458 subjects had a history of *H. pylori* eradication therapy. The infection had been successfully eradicated in 440 subjects (14.6%: eradicated negative). As shown in Table 2, the eradication therapy had failed in 18 subjects (eradicated positive), giving an eradication rate of 96.1% (440/458).

**Table 1:** Characteristics of Japanese subjects presenting for medical checkups (*n* = 3,005) evaluated by upper gastrointestinal endoscopy

Age (years)	54.2 ± 9.1
Gender (males: females)	1,549: 1,456
Body mass index (kg/m^2^)^a^	22.8 ± 5.2
Number with *H. pylori* infection	894 (29.8%)
Number with successful eradication of *H. pylori*	440 (14.6%)
Number with hiatus herniation	888 (29.6%)
Number with Barrett’s esophagus	
Short segment	712 (23.7%)
Long segment	10 (0.3%)
Number with gastric ulcer scars	91 (3.0%)
Number with duodenal ulcer scars	77 (2.6%)

^a^Mean ± SD

**Table 2:** *Helicobacter pylori* infection and eradication therapy (numbers of subjects)

	History of eradication therapy	No eradication therapy
*H. pylori* positive	18 (eradicated positive)	876 (native positive)
*H. pylori* negative	440 (eradicated negative)	1,671 (native negative)

Table 3 shows the FSSG scores for reflux symptoms and acid-related dyspepsia compared between *H. pylori* positive and negative subjects. The symptoms evaluated by the FSSG were compared between two groups: an *H. pylori* native negative group versus an *H. pylori* native positive group. Age and body mass index (BMI) were not different between the groups, and other subject-related characteristics were not different between the groups. The score for acid-related dyspepsia was not different between the *H. pylori* native negative and positive groups, indicating that *H. pylori* infection had no influence on dyspepsia symptoms in subjects presenting for medical checkups. In contrast, the mean score for reflux symptoms evaluated by the FSSG was significantly higher in the *H. pylori* native negative group compared with the native positive group (*p* < 0.05). However, greater response with score reduction 2 or more for each question regarding reflux symptoms (Q1–Q7) were not different between *H. pylori* native negative and positive groups (data not shown).

**Table 3:** Comparison of total scores from FSSG questionnaire for reflux symptoms and acid-related dyspepsia, between *Helicobacter pylori* negative and positive subjects

	*H. pylori* native negative (*n* = 1,671)	*H. pylori* native positive (*n* = 876)	*p* value
Acid-related dyspepsia^a^	1.69 ± 2.67	1.64 ± 2.37	0.62
Reflux symptoms^a^	2.10 ± 2.87	1.85 ± 2.59	0.03
Age (year)^a^	54.3 ± 7.9	54.0 ± 9.3	0.22
BMI (kg/m^2^)^a^	22.9 ± 6.2	22.8 ± 3.3	0.61

	Eradicated negative (*n* = 440)	*H. pylori* native positive (*n* = 876)	*p* value
Acid-related dyspepsia^a^	1.63 ± 2.38	1.64 ± 2.37	0.96
Reflux symptoms^a^	1.78 ± 2.45	1.85 ± 2.59	0.60
Age^a^	54.4 ± 7.8	54.0 ± 9.3	0.48
BMI^a^	23.1 ± 3.5	22.8 ± 3.3	0.13

*FSSG* frequency scale for the symptoms of gastroesophageal reflux disease, *BMI* body mass index

^a^Mean ± SD

Reflux symptoms and acid-related dyspepsia were also compared between the two groups: subjects for whom *H. pylori* eradication was successful (eradicated negative) versus subjects who were infected with *H. pylori* and never received eradication therapy (native negative). As indicated in Table 3 (the lower low), age and BMI were not different between the two groups. The acid-related dyspepsia score for the successful eradication subjects (eradicated negative) was not better compared with that for the *H. pylori* positive subjects (native positive). Reflux symptoms in the successful eradication subjects (eradicated negative) were not exacerbated compared with *H. pylori* positive subjects (native positive), as the reflux symptoms scores were almost the same in these two groups.

Table 4 shows the effect of the time span after eradication therapy for *H. pylori*. The time span was not detected for 52 out of 440 eradicated subjects. As shown, neither the score for reflux symptoms nor the score for acid-related dyspepsia depended on the time span after eradication, whereas we did not follow up the same subjects as the present report was a cross-sectional study.

**Table 4:** Effects of the time span after eradication on FSSG scores

	Less than 3 years after eradication (*n* = 128)	More than 3 years (*n* = 260)	*p* value
Acid-related dyspepsia^a^	1.57 ± 2.08	1.81 ± 2.58	0.10
Reflux symptoms^a^	1.70 ± 2.53	1.78 ± 2.40	0.76
Age^a^	54.0 ± 8.2	54.3 ± 7.8	0.71
BMI^a^	22.9 ± 3.2	23.0 ± 3.8	0.83

*FSSG* frequency scale for the symptoms of gastroesophageal reflux disease, *BMI* body mass index

^a^Mean ± SD

## Discussion

The present study indicated that *H. pylori* infection had no influence on acid-related dyspepsia in relatively healthy Japanese subjects presenting for medical checkups. *H. pylori* infection is a major cause of peptic ulcers and gastric cancer, but this study excluded these diseases using upper gastrointestinal endoscopy. The subjects in the present study had fewer clinical symptoms compared with patients who visit a hospital, which might be one of the reasons there were no differences in dyspepsia between *H. pylori* positive and negative subjects as most of *H. pylori* related dyspeptic patients defined by Roma IV criteria [26] was excluded from the present study. Moreover, most previous Japanese studies have similarly suggested that there is no relationship between upper gastrointestinal symptoms (dyspepsia) and *H. pylori* infection [5–12].

The total score for reflux symptoms was higher in the *H. pylori* negative group compared with the *H. pylori* positive group. Whereas subjects with Los Angles classification grade A–D reflux esophagitis were not included in the present study, those subjects with grade M or N [12, 27, 28] were included. This might be a cause of higher scores for reflux symptoms. It is also possible that reflux symptoms might be more common in relatively healthy *H. pylori* negative subjects compared with similar *H. pylori* positive subjects in Japan.

Eradication of *H. pylori* had no influence on upper gastrointestinal symptoms in the present study. Regarding this eradication, the data were evaluated retrospectively, and the eradication itself and the time span after eradication changed neither the reflux nor the dyspepsia scores from the modified FSSG questionnaire. Previous studies indicated that endoscopic reflux esophagitis was developed after eradication [4, 15–20]. This study excluded endoscopic reflux esophagitis, and the upper gastrointestinal symptoms assessed by the FSSG were not affected by *H. pylori* eradication in subjects without reflux esophagitis. Previous reviews suggested that the time span after eradication might be important for the clinical symptoms [16, 18, 27]. Namely, reflux symptoms appeared after the eradication might be diminished in a time dependent manner after the eradication. The present study indicated the time span after eradication had no effect on the score of FSSG whereas this cross-sectional study did not follow up the same subjects.

In summary, the present study indicated that *H. pylori* infection did not affect upper gastrointestinal symptoms in relative healthy Japanese subjects presenting for medical checkups with no significant upper gastrointestinal lesions.
